# The Role of Haemoglobin Variants and Red Blood Cell Polymorphisms in Asymptomatic Malaria: A Cross‐Sectional Study

**DOI:** 10.1002/hsr2.72275

**Published:** 2026-04-02

**Authors:** Enoch Aninagyei, Comfort Addo Boatey, John Gameli Deku, Adjoa Agyemang Boakye, Ebenezer Nyarkotey, Gifty Larbi, Desmond Omane Acheampong

**Affiliations:** ^1^ Department of Biomedical Sciences, School of Basic and Biomedical Sciences University of Health and Allied Sciences Ho Volta Region Ghana; ^2^ Department of Medical Laboratory Sciences, School of Allied Health Sciences University of Health and Allied Sciences Ho Volta Region Ghana; ^3^ Department of Biomedical Sciences, School of Allied Health Sciences University of Cape Coast Cape Coast Central Region Ghana

**Keywords:** ABO blood groups, asymptomatic malaria, haemoglobin variants, red cell polymorphs

## Abstract

**Background and Aims:**

Human phenotypes, particularly blood group polymorphisms (ABO) and haemoglobin variants (HbAA and HbAS), have been widely studied in relation to clinical malaria. However, their role in asymptomatic malaria remains underexplored. This study therefore, investigated the association of these phenotypic variants with the burden of asymptomatic malaria among Ghanaians.

**Methodology:**

Community members without clinical signs of malaria were recruited from four endemic communities. Whole blood samples were screened for *Plasmodium falciparum* histidine‐rich protein 2 (PfHRP2). ABO blood group and Rhesus D factor typing were then performed, followed by confirmation of haemoglobin phenotypes using alkaline electrophoresis (pH 8.6). A multivariate logistic regression model was applied to assess the association between asymptomatic malaria and phenotypic factors, while adjusting for potential confounders.

**Results:**

Haemoglobin variants (*p* = 0.021) and red cell polymorphisms (*p* < 0.001) were significantly associated with asymptomatic malaria, whereas RhD types were not (*p* = 0.877). However, a significant association was observed when RhD types were combined with red cell polymorphisms (*p* = 0.00009). Individuals with haemoglobin AA had significantly higher odds of asymptomatic malaria (AOR = 2.1, *p* = 0.02). Although higher odds were observed among individuals with O Rh D–positive (AOR = 2.39), Group O/HbAA (AOR = 1.15), and Group B/HbAS (AOR = 1.22), these associations were not statistically significant.

**Conclusion:**

This study found a high prevalence of asymptomatic malaria among community members. Haemoglobin variants and ABO blood group polymorphisms were associated with asymptomatic malaria, with HbAA showing significantly higher odds of infection. Although higher odds were observed among individuals with O Rh D–positive, O/HbAA, and B/HbAS phenotypes, these associations were not statistically significant. These findings highlight the complex role of host genetic factors in asymptomatic malaria carriage and underscore the need for further studies with larger sample sizes to clarify these associations.

## 1 Introduction

Malaria control programmes have traditionally focused on symptomatic malaria [1]. However, to achieve the goal of malaria elimination, endemic countries must also address asymptomatic malaria. Although clinically silent, asymptomatic infections contribute to sustained transmission and may undermine elimination efforts [2]. Host genetic factors are known to influence susceptibility to malaria, with haemoglobin variants and red blood cell (RBC) polymorphisms among the most studied. Haemoglobinopathies such as sickle cell trait (HbAS) [3, 4, 5] and α‐thalassaemia [6] are associated with partial protection against malaria. For example, individuals carrying HbS or HbC often harbour lower parasite densities than those with normal haemoglobin (HbAA). The protective mechanism is not fully understood, but evidence suggests that structural alterations in the *P. falciparum* erythrocyte membrane protein 1 (PfEMP1) may reduce the ability of infected RBCs (iRBCs) to adhere to host endothelial receptors, thereby limiting parasite survival and disease severity [7, 8].

ABO blood group polymorphisms have also been shown to influence malaria risk. Compared with blood group O, individuals with blood group A have been reported to be 16–17.8 times more likely to develop severe malaria [9]. Other non‐O groups, including B and AB, similarly increase vulnerability [10], largely due to enhanced rosetting and cytoadherence of iRBCs [11]. In contrast, Rhesus types have not been associated with clinical malaria [9].

Given these observations, the role of haemoglobin variants and RBC polymorphisms in malaria susceptibility is well recognised. However, their contribution to asymptomatic malaria remains poorly understood. This study, therefore, aimed to compare the burden of asymptomatic malaria among individuals with HbAA vs. HbAS, and across different ABO and Rhesus blood groups.

## 2 Materials and Methods

### 2.1 Study Design, Study Sites, Study Population, and Prevalence of Asymptomatic Malaria in the District

This cross‐sectional study was conducted in the Fanteakwa South District of the Eastern Region of Ghana, where the prevalence of asymptomatic malaria is estimated at 43.4% [12]. Participants were recruited between June and October 2023 from four randomly selected communities: Dwenase, Bososu, Nsutam, and Osino. Only community members without clinical signs of malaria at the time of selection were included. The prevalence of asymptomatic malaria in these communities was 36.9%, 27.7%, 50.0%, and 58.8%, respectively [12].

### 2.2 Sample Size Determination

Using Cochrane's formula, *n* = *z* ^2^ *p*(1 − *p*)/*d* ^2^, where *n* is the sample size, *z* is the confidence level at 95% (standard value of 1.96), *d* is the margin of error at 5% (standard value of 0.05), and the prevalence of asymptomatic malaria in the district was 43.4% [9], the minimum sample size of 377 was estimated for the study.

### 2.3 Inclusion and Exclusion Criteria

Participants were community members who presented without fever, chills, or headache at the time of sample collection. Individuals with a history of malaria or completion of malaria treatment within the preceding 60 days were excluded. Participants who reported self‐medication for malaria, either with herbal remedies or orthodox drugs, were also excluded.

### 2.4 Participant Selection and Blood Sample Collection

Details of participant selection have been published elsewhere [9]. Following recruitment, 2 mL of whole blood was collected from each participant. Venipuncture sites were disinfected with 70% alcohol followed by povidone‐iodine. For participants without palpable veins, particularly children, capillary blood was collected instead. All blood samples were placed in tubes containing CPDA‐1 anticoagulant [9].

### 2.5 Laboratory Procedures

#### 2.5.1 Detection of *P. falciparum* Histidine‐Rich Protein 2 (PfHRP2)

Detection of *P. falciparum* HRP2 was performed using the First Response malaria rapid diagnostic test kit (Premier Medical Corporation Pvt. Ltd., Gujarat, India). For each test, 5 µL of whole blood was dispensed into the sample window, followed by two drops of buffer into the buffer window. Results were read at 20 min. A test showing two red bands, at both the control and test lines, was interpreted as positive, whereas a single band at the control line indicated a negative result. Tests lacking a control band were considered invalid and repeated until a valid result was obtained.

#### 2.5.2 Determination of Red Cell Polymorphs

ABO blood group and Rhesus factor typing were performed using Accucare ABO/Rh monoclonal antibodies (Chennai, India) with the tile method. Briefly, 50 µL of anti‐A or anti‐B reagent was mixed with 20 µL of whole blood and gently agitated for approximately 30 s. The presence of visible agglutination indicated a positive reaction for the respective antigen, while its absence was considered negative. Blood samples of known groups (A Rh D positive, B Rh D positive, and O Rh D negative) served as controls.

#### 2.5.3 Determination of Sickle Cell Statuses

Sickle cell status was determined using 2% (w/v) sodium metabisulphite (Molar mass = 190.11 g/mol). Briefly, 2 μL of CPDA‐anticoagulated blood was mixed with 50 μL of the reagent on a microscope slide, covered with a coverslip, and incubated at room temperature for 30 min. Samples were examined microscopically at ×40 objective magnification, and the presence of sickled red blood cells was recorded. A known HbAS sample served as the positive control, and an HbAA sample served as the negative control.

#### 2.5.4 Phenotyping for Haemoglobin Variants

Haemoglobin phenotypes were determined using cellulose acetate alkaline electrophoresis at pH 8.6. Haemolysates were prepared by saline‐washing red blood cells, followed by lysis with 0.5% (v/v) Triton X‐100 in 100 mg/L potassium cyanide. Electrophoresis was performed on cellulose acetate membranes pre‐soaked in Tris‐EDTA‐borate (TEB) buffer (pH 8.4–8.6) under constant current (350 V, 50 mA) for 25 min. A positive control containing haemoglobins C, S, F, and A was included with each batch.

#### 2.5.5 Data Analyses

Data cleaning and validation were performed to identify and correct errors or inconsistencies. Descriptive statistics, including frequencies, percentages, means, and standard deviations, were used to summarise participants' sociodemographic characteristics. Inferential analyses were conducted to examine associations between red cell polymorphisms, haemoglobin variants, and malaria status. The Chi‐square test was used to assess differences between categorical variables. Multivariate logistic regression models were used to evaluate the associations between haemoglobin variants and red cell polymorphisms and malaria, while adjusting for potential confounders, namely, age, sex, educational status, occupation, and geographical location of the study participant. Separate multivariate logistic regression models were fitted for each genetic factor (ABO group, Rh factor, and haemoglobin genotype) to estimate their independent associations with asymptomatic *P. falciparum* infection, adjusting for age, sex, education, and occupation. Variables were not entered simultaneously to avoid collinearity between red blood cell polymorphisms. Associations between categorical variables were assessed using the Chi‐square test or Fisher's exact test, as appropriate. Variables with *p* < 0.05 in bivariate analysis were considered for inclusion in the multivariate logistic regression model to identify factors independently associated with asymptomatic *P. falciparum* infection. Adjusted odds ratios (AORs) with 95% confidence intervals (CIs) were calculated. All statistical tests were two‐sided, and a *p*‐value < 0.05 was considered statistically significant. All analyses were conducted using SPSS version 25 (IBM Corp., Armonk, NY, USA).

## 3 Results

### 3.1 Demographic Characteristics of the Study Participants

In total, 383 participants were recruited. Twelve samples were unsuitable for blood grouping due to haemolysis. Data from 15 additional samples were excluded because they contained results for either blood grouping or haemoglobin variants, but not both. Four more samples were rejected because their volumes were too small to allow confirmation by haemoglobin electrophoresis. Thus, data from 352 participants were valid for analysis. Of these, 91 (25.9%), 96 (27.3%), 85 (24.1%), and 80 (22.7%) were from Bososu, Dwenase, Nsutam, and Osino, respectively. The gender distribution was 40.1% (141/352) male and 59.9% (211/211) female. The largest age group was 0–19 years (49.7% [175/352]), while participants older than 79 years represented the smallest group (1.1% [4/352]). Regarding education, 47.2% (166/352) had completed some level of formal schooling, 3.7% (13/352) had no formal education, 11.9% (42/352) were below preschool age, and 37.2% (131/352) were still in school. More than half of the participants (56.3%) were unemployed (including minors and unemployed adults), while 36.1% were engaged in non‐farming occupations and 7.7% in farming. The majority were Christians (89.8%), with Muslims (8.2%) and Traditional worshippers (2.0%) forming the remainder (Table 1).

**Table 1:** Demographics, prevalence, and associations of asymptomatic malaria.

		Asymptomatic malaria status		
Variables	Total (%)	Positive (%)	Negative (%)	*p*‐value
Study site				0.15
Bososu	91 (25)	39 (42.9)	52 (57.1)	
Dwenase	96 (27.3)	33 (34.4)	63 (65.6)	
Nsutam	85 (24.1)	42 (49.4)	43 (50.6)	
Osino	80 (22.7)	39 (48.8)	41 (51.2)	
Gender				0.21
Male	141 (40.1)	67 (48.2)	74 (51.8)	
Female	211 (59.9)	86 (40.8)	125 (59.2)	
Age range (yrs)				0.031
0–19	175 (49.7)	90 (51.4)	85 (48.6)	
20–39	106 (30.1)	34 (32.1)	72 (67.9)	
40–59	43 (12.2)	18 (41.9)	25 (58.1)	
60–79	24 (6.8)	9 (37.5)	15 (62.5)	
> 79	4 (1.1)	2 (50.0)	2 (50.0)	
Formal education				< 0.001
Below preschool	42 (11.9)	13 (30.9)	29 (69.1)	
Completed a level	166 (47.2)	60 (36.1)	106 (63.9)	
Schooling	131 (37.2)	77 (58.8)	54 (41.2)	
No formal education	13 (3.7)	3 (23.1)	10 (76.9)	
Occupation category				0.003
Farming related	27 (7.7)	9 (33.3)	18 (66.7)	
Non‐farming related	127 (36.1)	39 (32.5)	81 (67.5)	
Unemployed	198 (56.1)	105 (51.2)	100 (48.8)	
Nature of occupation				0.001
Work both day and night	36 (23.7)	10 (28.6)	25 (71.4)	
Day only	112 (73.7)	38 (34.9)	71 (65.1)	
Night only	4 (2.6)	0 (0)	4 (100.0)	
Religion				0.69
Christian	316 (89.8)	139 (43.9)	177 (56.1)	
Muslim	29 (8.2)	12 (41.4)	17 (58.6)	
Traditional	7 (2.0)	22 (81.5)	5 (18.5)	

### 3.2 Prevalence and Factors Associated With Asymptomatic Malaria

The overall prevalence of asymptomatic malaria in the district was 43.5% (153/352). Point prevalence varied across study sites, with Nsutam recording the highest positivity rate at 49.4% (42/85) and Dwenase the lowest at 34.4% (33/96), though this difference was not statistically significant (*p* = 0.15). Females had a higher prevalence (56.2%) than males (43.7%), but the difference was also not significant (*p* = 0.21). Participants aged 0–19 years had a significantly higher prevalence of asymptomatic malaria (51.4%) compared with other age groups (*p* = 0.0312). Educational status (*p* < 0.001) and occupation (*p* = 0.003) were significantly associated with asymptomatic malaria. The highest prevalence was observed among participants currently in school (58.8%) and among those engaged in daytime‐only work (34.9%) (Table 1).

### 3.3 Impact of ABO and Rhesus Blood Group Polymorphisms on Asymptomatic Malaria

Among the participants, the most common haemoglobin variant was HbAA (85.2%), followed by HbAS (14.8%). With respect to ABO distribution, blood group O was the most prevalent (56.0%), followed by A (21.0%), B (19.6%), and AB (3.4%), while the majority of participants were Rhesus positive (91.8%) (Table 2). A significant association was observed between haemoglobin variants and asymptomatic malaria, with individuals carrying HbAA accounting for a higher proportion of asymptomatic malaria cases compared with those with HbAS (*p* = 0.021). ABO blood group polymorphisms were also significantly associated with asymptomatic malaria (*p* < 0.001), with blood group O contributing the largest proportion of positive cases. In contrast, Rhesus factor alone was not significantly associated with asymptomatic malaria (*p* = 0.88). When ABO blood groups were considered together with Rhesus types, a significant association with asymptomatic malaria was observed (*p* < 0.001).

**Table 2:** Haemoglobin variants and red blood cell polymorphisms in relation to asymptomatic malaria.

		Asymptomatic malaria status		
Variable	Total (%)	Positive (%)	Negative (%)	*p*‐value
Haemoglobin variants				0.021
AA	300 (85.2)	138 (90.2)	162 (81.4)	
AS	52 (14.8)	15 (9.8)	37 (18.6)	
Red blood cells polymorphs				
ABO groups				< 0.001
A	74 (21.0)	27 (17.6)	47 (23.6)	
B	69 (19.6)	16 (10.5)	53 (26.63)	
O	197 (56.0)	106 (69.3)	91 (45.73)	
AB	12 (3.4)	4 (2.6)	8 (4.02)	
Rhesus (Rh) types				0.88
Positive	323 (91.8)	140 (91.8)	183 (92.0)	
Negative	29 (8.2)	13 (8.2)	16 (8.0)	
ABO/Rh types				< 0.001
A positive	67 (19.0)	22 (14.4)	45 (37.959)	
B positive	64 (18.2)	16 (10.5)	48 (26.23)	
O positive	180 (51.1)	98 (64.1)	82 (44.81)	
AB positive	12 (3.4)	4 (2.6)	8 (4.37)	
A negative	7 (2.0)	5 (3.2)	2 (12.5)	
B negative	5 (1.4)	0 (0)	5 (31.25)	
O negative	17 (4.8)	8 (5.2)	9 (56.25)	

### 3.4 Combined Effect of ABO Blood Groups and Haemoglobin Variants on Asymptomatic Malaria

Table 3 shows the association between combined ABO blood group polymorphisms and haemoglobin variants with asymptomatic malaria. Among participants with normal haemoglobin (HbAA), the prevalence of asymptomatic malaria was significantly higher in blood group O (54.7%) (*p* = 0.001). Similarly, among those with HbAS, asymptomatic malaria prevalence was also higher in blood group O (48.1%) (*p* = 0.016). Rhesus type, however, showed no significant association with asymptomatic malaria, irrespective of haemoglobin status.

**Table 3:** Asymptomatic malaria prevalence by ABO blood group–haemoglobin variant combinations.

		Asymptomatic malaria status		
Variable	Total	Positive (%)	Negative (%)	*p*‐value
ABO/HbAA				0.001
Group A/HbAA	64	26 (17.0)	38 (83.0)	
Group B/HbAA	57	15 (26.3)	42 (73.7)	
Group O/HbAA	170	93 (54.7)	77 (45.3)	
Group AB/HbAA	9	4 (44.4)	5 (55.6)	
ABO/HbAS				0.016
Group A/HbAS	10	1 (10.0)	9 (90.0)	
Group B/HbAS	12	1 (8.3)	11 (91.7)	
Group O/HbAS	27	13 (48.1)	14 (51.9)	
Group AB/HbAS	3	0 (0)	3 (100)	
Rhesus/HbAA				0.73
Positive	272	126 (46.3)	146 (53.7)	
Negative	28	12 (42.9)	16 (57.1)	
Rhesus/HbAS				0.11
Positive	51	14 (27.5)	37 (72.5)	
Negative	1	1 (100)	0 (0)	

### 3.5 Predictive Role of Haemoglobin Variants and Red Blood Cell Polymorphisms in Asymptomatic Malaria

Multivariate logistic regression analysis indicated that individuals with haemoglobin variant AA had significantly higher odds of asymptomatic malaria (AOR = 2.10, 95% CI: 1.11–3.99). Blood group O was associated with reduced odds of asymptomatic malaria compared with non‐O groups (AOR = 0.26, 95% CI: 0.14–0.48). In subgroup analyses, higher odds of asymptomatic malaria were observed among individuals with blood group O Rhesus D positive (AOR = 2.39, 95% CI: 0.70–8.22) and those with the combined phenotype O/HbAA (AOR = 1.15, 95% CI: 0.39–5.82), although these associations were not statistically significant. Similarly, among individuals with HbAS, blood group B showed higher odds of asymptomatic malaria (AOR = 1.22, 95% CI: 0.16–9.56), but this association did not reach statistical significance (Table 4).

**Table 4:** Regression analysis of ABO/haemoglobin variants as predictors of asymptomatic malaria.

	Asymptomatic malaria status			AOR (95% CI)
Variable	Positive (%)	Negative (%)	*p*‐value	
Haemoglobin variants			0.02	
AA	138 (90.2)	162 (81.40)		2.1 (1.11–3.99)
AS	15 (9.8)	37 (18.59)		Ref
Red blood cell polymorphs				
ABO groups			< 0.001	
A	27 (17.6)	47 (23.6)		0.43 (0.13–1.47)
B	16 (10.45)	53 (26.63)		0.49 (0.29–0.86)
O	106 (69.28)	91 (45.73)		0.256 (0.14–0.48)
AB	4 (2.61)	8 (4.02)		Ref
ABO/Rh positive			< 0.001	
A positive	22 (15.71)	45 (37.959)		0.97 (0.27–3.60)
B positive	16 (27.74)	48 (26.23)		0.67 (0.012–2.51)
O positive	98 (70)	82 (44.81)		2.39 (0.70–8.22)
AB positive	4 (2.86)	8 (4.37)		Ref
ABO/Rh negative			0.047	
A negative	5 (38.46)	2 (12.5)		Ref
B negative	0 (0)	5 (31.25)		0.00
O negative	8 (61.54)	9 (56.25)		0.36 (0.053–2.37)
ABO/HbAA			0.002	
Group A/HbAA	26 (18.84)	38 (23.46)		0.86 (0.21–3.49)
Group B/HbAA	15 (10.87)	42 (25.93)		0.45 (0.11–1.89)
Group O/HbAA	93 (67.39)	77 (47.53)		1.15 (0.39–5.82)
Group AB/HbAA	4 (2.89)	5 (3.08)		Ref
ABO/HbAS			0.016	
Group A/HbAS	1 (6.67)	9 (24.32)		Ref
Group B/HbAS	1 (6.67)	11 (29.73)		1.22 (0.16–9.57)
Group O/HbAS	13 (86.67)	14 (37.83)		0.11 (0.03–0.57)
Group AB/HbAS	0 (0)	3 (8.11)		0.00

## 4 Discussion

In this study, 43.5% of participants were found to have asymptomatic malaria, indicating a substantial reservoir of silent *Plasmodium falciparum* infection within the community. This prevalence is comparable to reports from other malaria‐endemic settings in sub‐Saharan Africa, where asymptomatic parasitaemia remains common and contributes significantly to sustained transmission [2]. Although point prevalence varied across the study sites, with Nsutam recording the highest rate and Dwenase the lowest, these differences were not statistically significant, suggesting relatively similar transmission intensity across the communities. Age was significantly associated with asymptomatic malaria, with individuals aged 0–19 years exhibiting the highest prevalence. This observation is consistent with previous studies showing that children and adolescents in endemic regions frequently harbour asymptomatic infections due to the gradual acquisition of partial immunity following repeated exposure to malaria parasites [2]. In contrast, differences in prevalence between males and females were not statistically significant, indicating that sex did not play a major role in asymptomatic malaria carriage in this population.

Clearly, not all individuals living in endemic areas develop asymptomatic infection, underscoring the need to examine host‐related factors that may influence parasite carriage. In this regard, we investigated the role of human red blood cell polymorphisms, particularly ABO blood groups and haemoglobin variants, in shaping the risk of asymptomatic malaria.

Both ABO polymorphisms [13] and haemoglobin (Hb) variants [14] have been widely studied in relation to symptomatic malaria, although their role in asymptomatic infections remains less well understood. Blood group O has been shown to confer protection against severe malaria, largely through reduced rosetting and cytoadherence [11]. Rosetting refers to the clustering of uninfected erythrocytes around parasitised cells, which accelerates parasite invasion [15]. Similarly, HbAS has been associated with reduced risk of clinical malaria, likely due to impaired parasite growth under low oxygen tension [5, 16, 17] and reduced expression of parasite virulence proteins such as PfEMP1, which mediate endothelial cytoadherence [7, 8].

Our findings showed that individuals with HbAA had significantly higher odds of harbouring *P. falciparum* asymptomatically compared with those with HbAS. Among asymptomatic carriers, HbAA predominated (90.2%), whereas HbAS was less common (9.8%). These findings are consistent with previous evidence indicating that haemoglobin variants containing HbS impair parasite survival and promote more efficient clearance of infected erythrocytes from circulation. Consequently, individuals with HbAS tend to experience lower parasite densities and reduced susceptibility to malaria infection, which may explain the lower prevalence of asymptomatic carriage observed in this group.

With respect to ABO blood groups, descriptive analysis indicated that blood group O accounted for the largest proportion of asymptomatic malaria cases. However, multivariate logistic regression demonstrated that blood group O was associated with significantly reduced odds of asymptomatic malaria compared with non‐O blood groups. This finding aligns with previous studies showing that individuals with non‐O blood groups are more susceptible to severe malaria. For example, Aninagyei et al. [12] reported that the risk of severe malaria was substantially higher in blood groups A and B compared with group O, a finding supported by other studies [18, 19, 20, 21, 22]. Enhanced rosetting and cytoadherence observed in non‐O blood groups contribute to this increased vulnerability [11, 23, 24, 25]. In contrast, the reduced rosetting associated with blood group O may limit parasite sequestration and disease severity.

Although blood group O appeared to contribute a large proportion of asymptomatic cases in the descriptive analysis, this likely reflects its high population frequency within the study population rather than increased susceptibility. Multivariate regression analysis accounts for potential confounders and therefore provides a more reliable estimate of the independent association between blood group polymorphisms and asymptomatic malaria.

Consistent with findings reported in clinical malaria [9], the Rhesus factor was not significantly associated with asymptomatic malaria in this study. Overall, these findings highlight the role of host genetic factors, particularly haemoglobin variants and ABO blood group polymorphisms, in influencing malaria susceptibility and carriage dynamics in endemic populations.

## 5 Limitations

Interpretation of the associations between ABO blood group and asymptomatic infection should be made cautiously, as the small number of AB individuals (*n* = 12) may have resulted in unstable estimates. Moreover, the predominance of blood group O in the population suggests that its apparent contribution to the asymptomatic reservoir reflects both population frequency and complex host–parasite dynamics, wherein protection from severe disease may coincide with greater likelihood of silent carriage. In addition, the type of electrophoretic analysis done in this study made it difficult to distinguish haemoglobin A from D. This is because haemoglobin D co‐migrates with HbA, therefore, the frequencies of HbA reported in this study may include HbD.

## 6 Conclusion

This study demonstrated a high prevalence of asymptomatic malaria (43.5%) among community dwellers in the study district, highlighting the substantial silent reservoir that sustains transmission in endemic settings. Host red blood cell polymorphisms were associated with asymptomatic malaria carriage. Individuals with haemoglobin AA had significantly higher odds of harbouring asymptomatic *P. falciparum* infection compared with those with HbAS. In contrast, blood group O was associated with reduced odds of asymptomatic malaria compared with non‐O blood groups. The Rhesus factor alone showed no significant association with asymptomatic malaria.

These findings underscore the role of host genetic factors, particularly haemoglobin variants and ABO blood group polymorphisms, in influencing malaria susceptibility and parasite carriage in endemic populations. Given the high prevalence of asymptomatic infection observed, strengthened surveillance and further studies are needed to better understand host–parasite interactions and their implications for malaria control and elimination strategies.

## Author Contributions

**Enoch Aninagyei:** conceptualisation, writing – original draft, writing – review and editing, supervision, resources, investigation, project administration. **Comfort Addo Boatey:** methodology, investigation, writing – review and editing. **John Gameli Deku:** conceptualisation, investigation, supervision, writing – review and editing. **Adjoa Agyemang Boakye:** methodology, investigation, supervision, writing – review and editing. **Ebenezer Nyarkotey:** validation, formal analysis, data curation, writing – review and editing. **Gifty Larbi:** methodology, investigation, supervision, writing – review and editing. **Desmond Omane Acheampong:** conceptualisation, supervision, resources, project administration.

## Funding

The authors have nothing to report.

## Disclosure

The lead authors Enoch Aninagyei and Desmond Omane Acheampong affirm that this manuscript is an honest, accurate, and transparent account of the study being reported; that no important aspects of the study have been omitted; and that any discrepancies from the study as planned (and, if relevant, registered) have been explained.

## Ethics Statement

Ethical approval was obtained from the University of Health and Allied Sciences (UHAS) Research Ethics Committee (UHAS‐REC A.10 [122] 23‐24). Written informed consent was obtained from all adult participants and from parents or guardians of minors before enrolment. Participation was entirely voluntary, and all data were handled in accordance with relevant ethical guidelines and confidentiality standards.

## Conflicts of Interest

The authors declare no conflicts of interest.

## Data Availability

All data supporting the findings of this study are available within the article.
